# Quality of Clinical Counseling About Lifestyle-Related Diseases: An Analysis of Daily Practice From the National Guard Primary Health Care Center at Jeddah, Saudi Arabia

**DOI:** 10.7759/cureus.31551

**Published:** 2022-11-15

**Authors:** Hani S Almugti, Nouf N AlShutayri, Estabraq Mahdi, Badeia A AlMarzooq, Arwa Y Almowalad, Haneen A Khouja, Shahad Alnfisah, Ghaidaa Murad, Abdulrahman S AlZhrani, Mohammed A Alfuraydi, Aseel S AlOtaibi, Ammar Alabdullatif, Saeed A AlZahrani, Adeeb A Almarzooq, Rudhab Fallata

**Affiliations:** 1 Primary Health Care, Ministry of National Guard - Health Affairs, King Abdullah International Medical Research Center, King Saud bin Abdul-Aziz University for Health Sciences, Jeddah, SAU; 2 Laboratory, Health Plus Private Hospital, Jeddah, SAU; 3 Respiratory Therapy, King Abdullah Medical City, Jeddah, SAU; 4 Ophthalmology, Aljaber Specialized Hospital, Alahsa, SAU; 5 Medicine, King Abdul-Aziz University, Jeddah, SAU; 6 Intensive Care, Althaghor Hospital, Jeddah, SAU; 7 Pharmacy, Hayat National Hospital, Unizah , SAU; 8 Laboratory Medicine, Tetra Lab, Jeddah, SAU; 9 Medicine, Al Baha University, Al Baha, SAU; 10 Medicine, Majmaah University, Majmaah, SAU; 11 Pharmacy, Imam Abdulrahman bin Faisal Hospital, Dammam, SAU; 12 Medicine, Al-Maarefa University, Riyadh, SAU; 13 Dentistry, Imam Abdulrahman bin Fisal University, Dammam, SAU; 14 Pharmacy, Al-Ahmadi Hospital, Al Madinah, SAU

**Keywords:** primary healthcare, quality of counseling, education, counseling, lifestyle

## Abstract

Background: Unhealthy lifestyle behaviors are the main reason for the worldwide epidemic of chronic diseases. Changes in lifestyle, such as physical activity, healthy diet, and non-smoking, require preventive strategies at the national and individual levels. Clinical counseling is one of these strategies which helps patients to be motivated and enhance their self-management. The present study aimed to improve clinical counseling practice at the National Guard Primary Health Care Center, Jeddah, Saudi Arabia.

Objective*:*To assess the quality of counseling from the patient's perspective in the primary care center of the National Guard (specialized polyclinic center) using a modified counseling quality instrument (CQI).

Subjects and methods*:* In a cross-sectional study and through a convenient sampling technique from the patients visiting the National Guard Primary Health Care Center (specialized polyclinic center), 358 patients completed a self-administered questionnaire regarding their experiences of clinical counseling. The Kruskal-Wallis test was used to record the statistically significant differences between the ordinal variable of participants' quality scores and their demographic characteristics.

Results: The mean age of participants was 35± 8 years, ranging between 22 to 69 years. Most of the participating patients were women (63%). Slightly more than half the patients obtained clinical counseling during their appointments, and physicians at the clinic gave 57% of these clinical counseling sessions. The patients were generally satisfied with the counseling. They rated the counseling sessions as good in terms of content, the process of counseling, the way of interaction, and the degree of goal-oriented discussion. In addition to receiving counseling from physicians, older participants and those with postgraduate education were found to significantly positively affect patient perceptions of counseling quality.

Conclusion:This study found that half of the participants did not get clinical counseling during their visits, indicating no standardization in clinical counseling services for all patients. Although patients were generally satisfied with the counseling session provided to them, time constraint was the highest dissatisfaction item among two-thirds of the patients in the present study.

## Introduction

The burden of non-communicable diseases (NCDs) is a global public health problem that jeopardizes socio-economic development [1]. Non-communicable diseases were responsible for the death of 41 million people worldwide, representing 71% of all causes of death [2]. This number is estimated to rise to 55 million by 2030, particularly in developing countries [2]. The global status report from the World Health Organization (WHO) indicated that deaths from NCDs are attributed mainly to cardiovascular diseases (48%), cancers (21%), chronic respiratory diseases (12%), and diabetes (3.5%) [3]. In Saudi Arabia, the annual mortality rate from NCDs is 753 deaths per 100,000, and cardiovascular diseases are responsible for 314 (37%) of all these deaths [3,4].

The most prevalent behavioral risk factors contributing to NCDs involve physical inactivity, unhealthy diets, tobacco use, and the harmful use of alcohol. Studies reported that 80% of heart disease, stroke, type 2 diabetes, and 40% of cancer could be prevented by addressing a healthy diet and lifestyle improvements [3]. To reduce the most premature deaths from NCDs, a healthcare system should respond effectively to the community's health needs by activating the lifestyle medicine practice as an effective clinical action plan [5].

Primary health care services grant access to higher-risk groups and have opportunities to provide a continuous and comprehensive type of care [6]. Several recommended interventions were addressed in the preventive medicine and public health field to tackle the rising death rate from NCDs, including community-based interventions and public health policies [7]. These efforts, however, should be backed up by clinical care at the individual level through the primary health care centers.

Counseling patients on lifestyle modification is a fundamental competency for primary healthcare physicians [8]. A large volume of studies stated that the most frequent barriers to providing satisfactory quality clinical counseling are the increased number of patients and the busy clinic time [9,10]. The quality of clinical counseling has gotten less attention, and there has been a growing interest in improving counseling services using a quick, simple model and patient-centered approaches [11-,2].

There is no clear definition of high-quality clinical counseling. According to previous research, the content, application, and positive outcomes, along with counseling materials or methods, all influence the quality of patient counseling [11,12]. A positive attitude toward NCDs and high adherence to the care plans after the clinical counseling were reported as positive outcomes of high-quality counseling in a previous study [13].

In the healthcare context, for improvement purposes, there is a need to assess any intervention using various data collection techniques, including structured and semi-structured data collection tools [14]. Researchers indicated some factors in determining the quality of counseling, including the patients' needs, the counselor's competencies, and a good understanding of the cultural and socio-economic risk factors [9,11].

However, there is limited research in Saudi Arabia about the quality of lifestyle clinical counseling from the patients' perspective. The present study aims to improve clinical counseling practice at the National Guard Primary Health Care Center in Jeddah, Saudi Arabia.

## Materials and methods

Aim of the study

The study aimed to improve the clinical counseling practice at the National Guard Primary Health Care Center in Jeddah, Saudi Arabia. The primary objective was to assess the quality of counseling from the patients' perspective using an adapted counseling quality instrument (CQI). The secondary objective was to identify the factors (demographic, social, medical, and job title of the counselor) that may influence the quality of counseling.

Study area/setting

The study was carried out in the outpatient clinics of the National Guard Primary Health Care Center (specialized polyclinic center). This center is the largest National Guard Primary Health Care Center in the western region in terms of capacity and number of trained medical staff. It was chosen for our study because it covers a big part of the National Guard population and has specialized clinics dedicated to preventing and controlling chronic diseases. 

Study design and subjects

This study is a cross-sectional study that recruited patients who were visiting the primary health care center and had the following inclusion criteria: older than 18 years, had a health check-up scheduled at a preventive medicine clinic, or have been diagnosed with NCDs and scheduled at a chronic disease clinic. We excluded patients visiting the primary health care center due to an emergency health condition.

Sample size and sampling technique

The target sample size was 385 participants at a 5% margin of error and confidence level of 95%; there are approximately 50,000 of the population (National Guard dependents) who are under the coverage of the National Guard Primary Health Care Center (specialized polyclinic center) with a daily appointment rate of 500 to 600. Through a convenient sampling technique (non-probability sampling), the study approached patients who met the inclusion and exclusion criteria using a self-administered questionnaire.

Data collection methods, instruments used, and measurements

Variables of the Study

The dependent variables were the scores of the quality of clinical counseling whereas the independent variables were age, gender, level of education, marital status, medical history of the participants, and the job type of the counselor (nurse/physician/health educator).

Quality of Clinical Lifestyle Counseling

The quality of clinical counseling was defined based on the CQI [15] that was designed to evaluate the quality of patient counseling through four domains i.e., the content of counseling, implementation of counseling, the benefit of counseling, and counseling materials and methods. The content of counseling includes the topic that will be addressed during counseling such as symptoms of the diseases, disease risk factors, and medication. Implementation of counseling includes the process that will be conducted, the way of interaction, and the degree of goal-oriented counseling. The benefit of counseling includes the level of knowledge of disease and practice that is considered to have positive outcomes after clinical counseling. Lastly, counseling materials and methods include time spent on counseling, counseling materials, and counseling tools.

Questionnaire

The questionnaire of this study was self-administered and consisted of two sections. The first section included the demographic data, the medical history of the participants, and the job type of the counselor (see Appendix A). The second section included the assessment items of clinical counseling into three domains i.e., the content of counseling, implementation or process of counseling, and benefit of counseling (see Appendix B). It was adapted from the CQI [15] and the evidence-based strategies for counseling patients in primary care [11]. To ensure face and content validity, the questionnaire and related questions were translated into Arabic and then to English again and were revised by an expert panel of specialists in family medicine, health education, health quality, and mental health. It was tested for reliability by measuring its internal consistency. The Cronbach alpha coefficient was 0.76, indicating good reliability. 

The responses for all the first two domains were measured using a scoring system ranging from one (poor) to three (excellent). The highest scores for the first and second domains were 18 and 24, respectively. Assessment grading scores of these two domains were calculated for each participant and categorized into three following categories: poor satisfaction if the calculated total score was less than or equal to 6 for the first domain and less than or equal to 8 for the second domain, good satisfaction if the calculated total score was from 7 to 12 for the first domain and from 9 to 16 for the second domain, excellent satisfaction if the calculated total score was more than 13 for the first domain and more than 16 for the second domain. The responses for the third domain were measured using the three options (no/not that much/yes); the highest score for the third domain was 15.

Data management and analysis plan

For data entry and statistical analysis, the SPSS statistical software version 20 (IBM Corp., Armonk, NY, USA) was used. Quality control was performed at the coding and data entry stages. Data were presented using descriptive statistics in the form of frequencies and percentages for qualitative variables and means and standard deviations for quantitative variables. The Kruskal-Wallis test was used to record the statistically significant difference between the ordinal variable of participants' quality scores and their demographic characteristics.

## Results

Characteristics of the study subjects

According to the study design, 385 patients were included. Their mean age was 35± 8 years, ranging between 22 to 69 years. Almost two-thirds of the participants (63%) were females, and one-third (38%) of all participants were single. Slightly more than half the participants had a bachelor's degree (57%), and only 5 % had a postgraduate level of education (Table 1).

**Table 1:** Demographic characteristics of participants (n=385) SD: Standard deviation

Demographic characteristics	Frequency	Percentage (%)
Age		
Range	22 to 69 years	
Median	34 years	
Mode	36	
Mean ± SD	35 ± 8 years	
Gender		
Male	142	37
Female	243	63
Level of Education		
High school	146	38
Bachelor degree	219	57
Master/PhD	19	5
Marital Status		
Single	150	39
Married	235	61

Medical history

**Figure 1:**
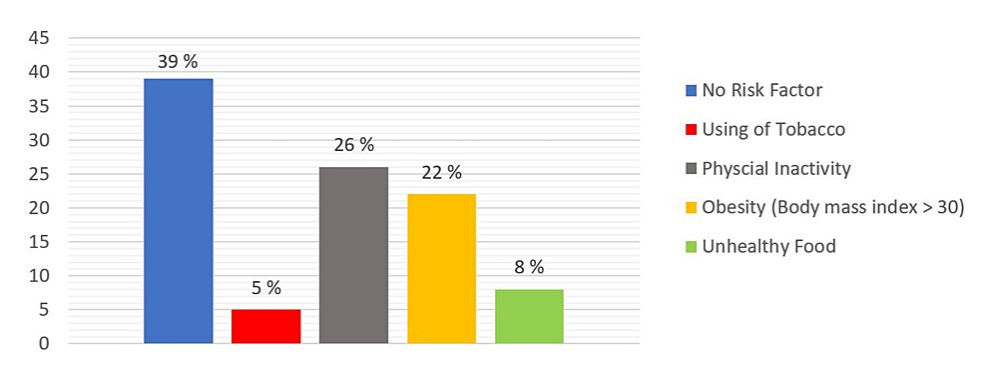
Percentages of participants with different health risk factors (n=385)

From the medical history, it was found that only 5 % of the participants were tobacco users. Almost one-third of them were physically inactive, and 22 % were obese. Only 8 % reported that they used to eat more bread, rice, and sweets than fruit and vegetable per week (Figure 1). 

**Figure 2:**
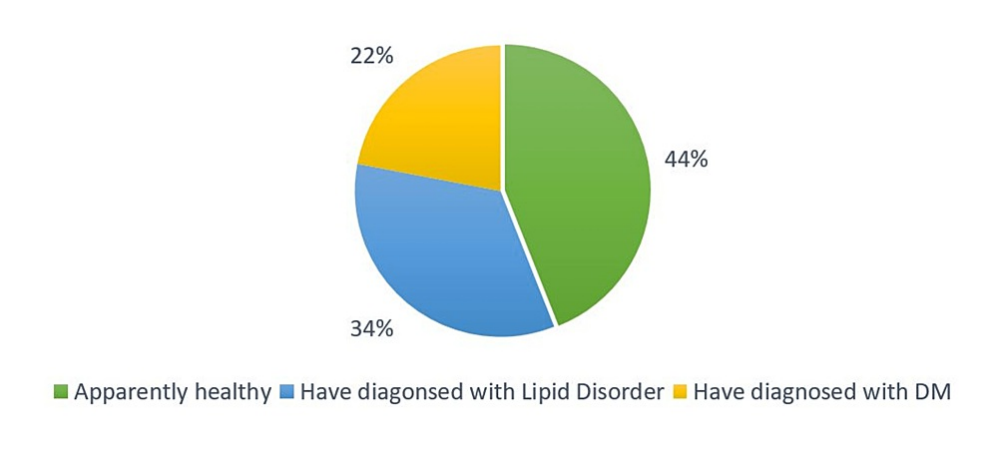
Percentage of participants who had lifestyle-related diseases (n=385)

Figure 2 indicates that almost half (44 %) of the participants reported that they were free from lifestyle-related chronic diseases, and a third of them (34 %) had been diagnosed with lipid disorders. However, diabetic participants constituted 22 % of all participants.

Assessment of clinical counseling

**Figure 3:**
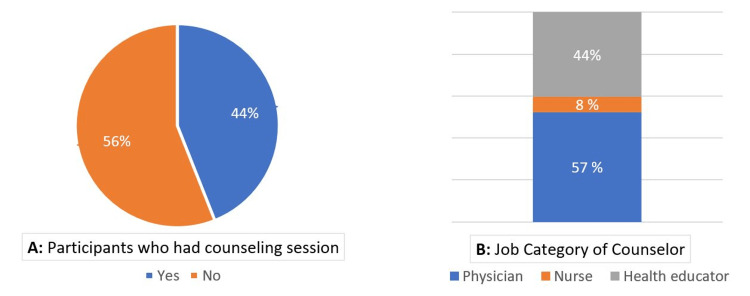
Percentage of participants who had a counseling session (A), and the different job categories of the counselor (B)

As shown in Figure 3, almost half of the participants (44%) obtained clinical counseling sessions during their visits to this primary healthcare facility. Slightly two-thirds (57%) of clinical counseling sessions were given by physicians.

**Figure 4:**
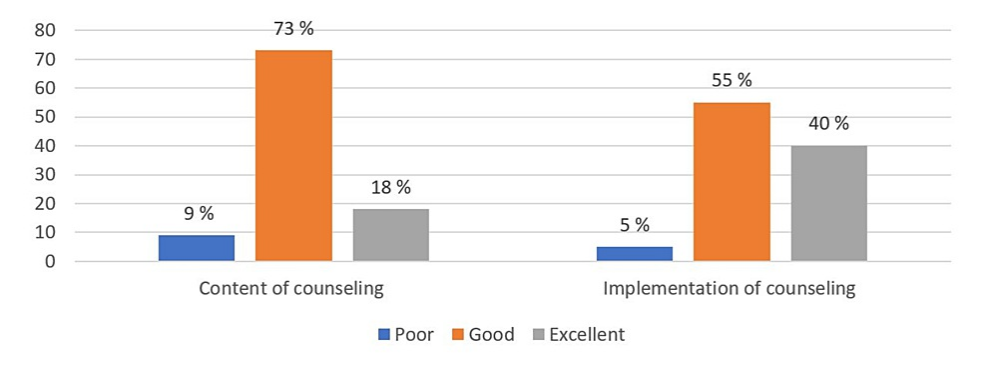
Participants' general assessment of the counseling session for the domains of content and implementation of counseling (n=169)

Concerning the clinical counseling assessment, Figure 4 shows that more than two-thirds of the participants rated the counseling sessions as good in terms of content, while their assessment grade scores were excellent by more than one-third of the participants (40 %) in terms of the process of counseling, way of interaction, and the degree of goal-oriented discussion.

Table 2 illustrates that at least two-thirds of participants rated all the assessment items of counseling content as good. At least one-third of participants agreed that they obtained an excellent discussion concerning the ideas that may help to overcome the barriers, possible ways to monitor the progress in lifestyle change, and the availability of a smoking cessation clinic or nutrition clinic. The median agreement scores were “2” indicating a good rating score.

**Table 2:** Participants' assessment of the content of counseling (n=169) SD: Standard deviation

Item	N (%)			Score (max=3)	
	Poor	Good	Excellent	Mean ± SD	Median
Effects of the current health risk factor on your health?	23 (14%)	124 (73%)	22 (13%)	1.85 ± 0.8	2
Importance of including lifestyle modification with medication to control your disease	25 (15%)	121 (72%)	23 (14%)	1.96 ± 0.7	2
Barriers to making a change in your lifestyle	22 (13%)	124 (73%)	23 (14 %)	1.88 ± 0.8	2
Ideas that may help to overcome the barriers	19 (11 %)	120 (71%)	30 (18%)	2 ± 0.7	2.1
Explain the ways to monitor the progress regarding your lifestyle change	21 (12.5%)	110 (65%)	38 (23%)	2.09 ± 0.7	2.2
Offering other supportive health services in the primary health care center can help change your lifestyle (smoking cessation clinic/nutrition clinic)	21 (12.5%)	117 (69%)	30 (18%)	1.92 ± 0.8	2

As regards the assessment of the counseling process, Table 3 indicates that at least half of the participants expressed a good perception of all the assessment items of the counseling process and implementation. Moreover, the median scores for these items were “2,” indicating a good rating score.

**Table 3:** Participants' assessment of the counseling process (n=169) SD: Standard deviation

Item	No (%)			Score (max=3)	
	Poor	Good	Excellent	mean±SD	Median
Use understandable words and clear sentences	8 (5%)	92 (54%)	69 (41%)	2.31 ± 0.7	2.2
Present the facts and concepts in a logical order	22 (13%)	93 (55%)	54 (32%)	2.06 ± 0.9	2
Uses appropriate counseling aids or education tools to support counseling	11 (6.5%)	105 (62%)	53 (31%)	2.22 ± 0.7	2
Verifies patient's understanding via feedback	8 (5%)	110 (65%)	51 (30%)	2.27 ± 0.7	2.1
Appropriate eye contact	20 (12%)	88 (52%)	61 (36%)	2.09 ± 0.6	2
Voice is audible; the tone and pace are good	6 (4%)	98 (58%)	65 (38%)	2.03 ± 0.7	2
Body language, postures, and gestures support the spoken message	8 (5%)	100 (59%)	61 (36%)	2.04 ± 0.6	2
Distance between you and the health care professional is appropriate	18 (11%)	108 (64%)	43 (25%)	2.01 ± 0.6	2

Table 4 demonstrates that 96% of the participants reported no need for another counseling session. In contrast, the lowest satisfaction was with the duration of counseling, where 63% of participants agreed the time was not enough for questions and discussion. On the other hand, half of the participants understood the importance of a healthy lifestyle in promoting their health and planned to share this information with their family or friends. However, two-thirds of participants (70 %) were ready to change their lifestyle after this session.

**Table 4:** Participants' perceptions and opinions regarding counseling benefits (n=169)

Item	N (%)		Score (max=3)		
	No/Not that much	Yes	Mean±SD	Median	
Do you feel this session helped you understand the importance of a healthy lifestyle in promoting health?	75 (44%)	94 (56%)	2.4 ± 0.5	2	
Do you plan to share this information with your family or friends?	79 (47%)	90 (53%)	2.5 ± 0.5	3	
Are you ready to change your lifestyle after this session?	50 (30%)	119 (70%)	2.7 ± 0.45	3	
Was the duration of the session long enough for questions and discussion?	107 (63%)	62 (37%)	2.3 ± 0.6	2	
Do you need another session?	161 (96%)	7 (4%)	1.7 ± 0.5	2	

Regarding the relation between participants' total assessment score and their demographic characteristics, Table 5 demonstrates higher median scores among older participants and those with a postgraduate education level; the differences were statistically significant (p < 0.05). However, the differences could not reach statistical significance with variables of gender and marital status.

**Table 5:** Relation between participants' total assessment score and their demographic characteristics (n=169) (*) Statistically significant at p<0.05

Demographic characteristics		
	First Domain: Content of counseling Assessment score (max=18)	Second Domain: Implementation of counseling Assessment score (max=24)
	Median	Median
Age		
<30 years	12	16
30 to 39 years	11	20
40 + years	14	21
Kruskal-Wallis test	1.12	2.2
p-value	0.019*	0.01*
Gender		
Male	12	16
Female	12	16
Kruskal-Wallis test	0.059	0.830
p-value	0.988	0.362
Educational level		
High school	12	16
Bachelor degree	12	16
Master/PhD	17	22
Kruskal-Wallis test	5.83	8.4
p-value	0.001*	0.001*
Marital status		
Single	12	16
Married	12	16
Kruskal-Wallis test	3.6	0.106
p-value	0.142	0.74

In multivariate analysis (Table 6), the education level of participants and the jobs of the counselor were identified as predictors of the benefits of counseling. The model indicates that both factors were statistically significant independent predictors. Thus, patients who had clinical counseling from physicians were more likely to get benefits from clinical counseling by four times compared with health educator staff. Meanwhile, patients with a postgraduate education level had a higher likelihood of benefitting from clinical counseling sessions three-fold compared with those with a high school level of education, and those with a bachelor's degree have only one-and-a-half times more likelihood of getting benefit from clinical counseling compared with participants with a high school level of education.

**Table 6:** Best-fitting multiple logistic regression models for the benefits of counseling B: Coefficient for the constant (also called the "intercept"), SE: Standard error, Wald: Wald chi-square test, df: Degrees of freedom, CI: Confidence interval, OR: Odds ratio

	B	SE	Wald		df	p-value		OR	95.0% CI for OR	
									Upper	Lower
Constant	- 1.902	0.905	4.43		1	0.035				
Job of the counselor (reference: health educator)										
Nurse	0.197	0.682	0.078		1	0.781	1.2		0.31	4.6
Physician	1.391	0.534	6.7		1	0.009	4.02		1.4	8.44
Education level of participant (reference: High school)										
Bachelor degree	0.450	0.274	2.700		1	0.100	1.569		0.917	2.684
Master/PhD	1.197	0.320	14.028		1	0.000	3.309		1.769	6.190
Nagelkerke R Square: 0.112										
Hosmer and Lemeshow Test: p=0.551										
Omnibus tests of model coefficients: p<0.001										

## Discussion

Patients' perceptions and satisfaction with health care services are valuable and frequently used as quality indicators in medical care. Researchers indicated that improved quality of care is associated with increased adherence to treatment processes and to the recommended preventive measures, which in turn leads to better health [16,17]. This study described patients' assessments of the clinical counseling at the National Guard Primary Health Care Center (specialized polyclinic) and aimed to improve the clinical counseling about lifestyle-related diseases, in addition to explaining factors associated with the quality of counseling.

Almost half the participants in the current study reported that they did not obtain clinical counseling during their medical visits. This finding matches those observed in earlier studies [18,19] that found that an increased number of patients with limited time devoted to each patient is a barrier to patient education and counseling. Another possible explanation for this finding is a variation of visits in primary healthcare as most patients present with multiple complaints and thus requiring physicians to divide time and resources during a visit to deal with competing demands. However, these challenges should not be an obstacle to providing standardized preventive services against common local preventable diseases. A previous study [18] suggested operational strategies for enhancing work capacity and leadership support to overcome these barriers and sustain the quality of health service.

According to the result of the present study, almost two-thirds of participants were satisfied with the clinical counseling session; these sessions were rated by them as good in terms of content and implementation process. However, two-thirds of participants expressed low satisfaction with the duration of counseling (the time was not enough for questions and discussion). This anticipated finding further supports the role of time constraints as a profession-related barrier affecting patient satisfaction. It is considered the highest dissatisfaction item among patients in a previous study [20].

Prior studies reported that patients' satisfaction differs based on their clinical and demographic characteristics. The education level of patients has been shown to be directly proportional to the health indicators, such as mortality and incidence of NCD [21,22]. The current study found that patients with a postgraduate education level had higher satisfaction and high assessment scores than those with a lower educational level. This figure seems consistent with other research, which found that highly educated patients are more able to communicate with their healthcare provider and thus resulting in improved adherence to treatment and a better outcome [22].

Moreover, patient age is another demographic factor affecting patient satisfaction. Earlier studies found that older patients generally seem more satisfied [23,24], which aligns with the current study's result that demonstrated relatively high assessment scores among older patients. A possible explanation for this might be related to the declined health status of older patients and the higher level of self-caring they have at this age. However, the medical history of patients was addressed as a confounder factor and independent variable in multivariate analysis in the current study, but the association with assessment score did not reach statistical significance.

The multivariate analysis in the current study identified the clinical counseling from physicians and participants with a higher level of education as positive factors increasing the benefits of the clinical counseling session. This might be attributed to the clinical pathway of primary health care clinics that dedicated more time to physicians, in addition to the high opportunities that physicians have from utilizing the medical history to encourage their patients to understand how lifestyle change is personally relevant to their clinical conditions. Although these results differ from some published studies [25,26], they are consistent with a previous study [27] that showed patient satisfaction with healthcare services provided by physicians was higher than the services by nurses. However, a collaborative teamwork system from nurses, physicians, and other health professionals has been implemented in different countries and is considered a practical solution for time constraints [27].

In terms of strengths, the questionnaire of this study was CQI, which showed adequate content validity in prior studies. Moreover, the validity of this modified questionnaire was also tested through factor analysis and by having an expert panel to evaluate the content of the questionnaire. Although the study has successfully demonstrated some clinical and demographic factors that may affect patients' satisfaction regarding clinical counseling, it has certain limitations in terms of generalizability-for instance, using the cross-sectional study design and representing only one primary care center as the study setting.

## Conclusions

This study aimed to improve lifestyle counseling at one of the National Guard's primary healthcare centers by assessing the quality of counseling from the patient's perspective, which is an indicator of their satisfaction with the daily practice. This study found there was no standardization in clinical counseling services, and almost half did not get a clinical counseling session during their appointments. Among those who had clinical sessions of counseling, the second major finding was a higher level of satisfaction with the content and implementation process of these sessions reported by almost two-thirds of them, thus indicating the good quality of the clinical counseling provided. Concerning the association between assessment scores and demographic factors, the older participants and those with a postgraduate education level were positively associated with higher assessment scores of clinical counseling quality 

## Appendices

Appendix A

First Section (Participant’s Characteristics)

o   Age

o   Gender: Male/Female

o   Level of education: illiteracy/below high school/high school/bachelor's/postgrad

o   Marital status: Single/married/divorced/widowed

o   Do you have any of the followings health risk factors (you can choose more than one item)

-         Obesity (BMI more than 30)

-         Use of tobacco (smoking )

-         Physical inactivity (physical activity less than two hours per week)

-         Unhealthy food (bread, rice, and sweets more than fruit and vegetables per week)

-         No, I don't have any

o   Have you been diagnosed with any of the following (you can choose more than one item)

-         Diabetes

-         Hypertension

-         Lipid disorder

-         Cardiovascular diseases

-         Cancer

-         Other: specify

-         No, I don't have any

o   Reason for visiting the primary health care center

-         Appointment (planned visit)

-         Emergency visit

Appendix B

Second Section (Assessment of the Clinical Counseling)

o   The first two questions are general and do not represent any domain used to be recorded separately from the remaining  questions:

a.      Question one: Do you get a counseling session about your health condition today? (yes/no)

b.      Question two: Who provides this session to you? (physician/nurse/health educator)

o   First domain: Content of counseling, including the following questions:

1)     Discuss the effects of your current lifestyle (obesity, smoking, High blood pressure, high lipid, bad diet, or physical inactivity) on your health.

2)     Explain the importance of including lifestyle modification with medication to control your disease.

3)     Discuss the barriers to making a change in your lifestyle.

4)     Discuss the ideas for overcoming the barriers to the lifestyle change you mentioned.

5)     Explain the ways to monitor the progress regarding your lifestyle change (follow-up appointment/lab parameters)

6)     Discuss the available health service in the primary health care center that can help to change your lifestyle (smoking cessation clinic/nutrition clinic).

o   Second domain: Implementation of counseling, including the following parameters

1)     Uses understandable words and clear sentences

2)     Presents the facts and concepts in a logical order

3)     Uses appropriate counseling aids or education tools to support counseling (e.g., poster/picture/video education model)

4)     Verifies patient's understanding via feedback

5)     Appropriate eye contact

6)     Voice is audible; the tone and pace are good

7)     Body language, postures, and gestures support the spoken message

8)     Distance between you and the health care professional is appropriate

The responses for the above two domains were measured using a scoring system ranging from one (poor) to three (excellent).

o   Third domain: Benefit of counseling, including the following questions

1)     Do you feel this session helped you understand the importance of a healthy lifestyle in your health?

2)     Do you plan to share this information with your family or friends?

3)     Are you ready to change your lifestyle after this session?

4)     Was the duration of the session long enough for questions and discussion?

5)     Do you need another session?

The responses for the above third domain were measured using the three options (no/not that much/yes).
